# Microscopic changes and gross morphology of placenta in women affected by gestational diabetes mellitus in dietary treatment: A systematic review

**DOI:** 10.1515/med-2025-1142

**Published:** 2025-02-13

**Authors:** Rossella Molitierno, Amalia Imparato, Nicola Iavazzo, Cecilia Salzillo, Andrea Marzullo, Antonio Simone Laganà, Andrea Etrusco, Vittorio Agrifoglio, Antonio D’Amato, Esposito Renata, Maria Giovanna Vastarella, Pasquale De Franciscis, Marco La Verde

**Affiliations:** Department of Woman, Child and General and Specialized Surgery, University of Campania “Luigi Vanvitelli”, 80138 Naples, Italy; Department of Precision and Regenerative Medicine and Ionian Area, Pathology Unit, University of Bari “Aldo Moro”, 70124 Bari, Italy; Department of Experimental Medicine, PhD Course in Public Health, University of Campania “Luigi Vanvitelli”, 80138 Naples, Italy; Unit of Obstetrics and Gynecology, “Paolo Giaccone” Hospital, Department of Health Promotion, Mother and Child Care, Internal Medicine and Medical Specialties (PROMISE), Unit of Obstetrics and Gynecology, “Paolo Giaccone” Hospital, University of Palermo, 90127 Palermo, Italy; Department of Interdisciplinary Medicine (DIM), Unit of Obstetrics and Gynecology, University of Bari “Aldo Moro”, Policlinico of Bari, 70124 Bari, Italy; Department of Environmental Biological and Pharmaceutical Sciences and Technologies, University of Campania “Luigi Vanvitelli”, Caserta, 81100, Italy

**Keywords:** gestational diabetes mellitus, diet therapy, placenta, placental pathology, gross morphology, microscopic morphology

## Abstract

**Introduction/objective:**

Gestational diabetes mellitus (GDM) influences adverse maternal and fetal outcomes. Nutritional therapy and exercise are the first steps to maintain normal glucose levels. During pregnancy, metabolic status influences placental development.

**Methods:**

This systematic review focused only on the morphology of the placenta and its microscopic changes in GMD under dietary therapy. A systematic search was performed on the main databases from inception to September 2024 (PROSPERO ID: CRD42024581621). Only original articles on GDM in diet and exercise treatment that reported at least one outcome of interest (microscopic features and macroscopic morphology of the placenta) were included.

**Results:**

A total of 716 studies were identified, and nine met the inclusion criteria. The analysis confirmed that despite dietary control, some morphological changes in the placenta, including villus immaturity, chorangiosis, and fibrinoid necrosis, occurred at a different rate. In addition, the included studies reported an increase in placental weight in the diet-controlled GDM group.

**Conclusion:**

Therefore, the results of the present qualitative analysis show that pregnant women with diet-controlled GDM, despite adequate glycemic control, abnormal placental development may persist. Our findings remark on the importance of the correct diet-managed GDM pregnancy monitoring due to the placental morphology abnormalities related to GMD.

## 1 Introduction

Gestational diabetes mellitus (GDM) is described as glucose intolerance with onset or first recognition during pregnancy [1]. This is one of the frequent pregnancy complications, and its prevalence is rising globally and reflects the increased prevalence of obesity and metabolic syndrome in whole populations [2]. Globally, the GDM prevalence ranges from 5 to 20% of all pregnancies and represent a critical public health issue [3–5]. This depends on varied diagnostic criteria, such as populations under study and practices used in screening [3–5]. Recent diagnostic criteria, like those identified by IADPSG, tend to recognize more cases [6]. GMD exposes both the mother and child to a high risk of developing long-term consequences later in life: a high risk of developing type 2 diabetes mellitus (T2DM) and cardiovascular diseases for the mother and a high risk of metabolic disorders and cardiovascular diseases in adulthood for the fetus [7,8]. Uncontrolled glycemia impacts maternal and fetal outcomes, with an increased risk of macrosomia, neonatal hypoglycemia, hyperbilirubinemia, dystocia of the shoulder, preterm delivery, primary cesarean section, and preeclampsia associated with GDM [9–14]. The complex etiologies of GDM include being overweight or obese, inadequate eating habits, high blood pressure, impoverished supply of proper micronutrients, endocrine dysfunctions, advanced maternal age, and family history of diabetes [15–19]. Correct management of the treatment of GDM may reduce these adverse outcomes [20]. Dietary management is the first line of treatment for cases of mild blood sugar elevation; exercise and nutritional therapy help to maintain glucose levels [21]. Medical nutrition therapy (MNT) for GDM highlights postprandial glucose control to reduce hyperglycemia and prevent adverse pregnancy outcomes [22]. MNT with low glycemic index have been found to be effective because these foods are digested and absorbed gradually and their absorption would result in a gradual increase in blood glucose levels [23]. MNT helps in the stabilization of blood glucose levels through adjustments in carbohydrate intake and adequate foods to achieve a reduced and more gradual absorption pattern [22]. Physical activity is promoted to reduce insulin resistance and support glycemic control [24]. In addition, ultra-processed food intake has been associated with a higher inflammatory potential in pregnancy, and this condition could also impact the maternal inflammatory status [25–28]. Nevertheless, some women, especially with higher baseline glucose levels, cannot achieve optimal glycemic control with MNT alone [22,23]. Dietary interventions represent a safe option to manage GDM, and MNT influences the maternal metabolism directly by lowering the peak postprandial blood glucose value, reducing the postprandial curve variation, and reducing systemic inflammation. Such benefits could contribute to the placenta function, reducing the hyperglycemia stress and related vascular abnormalities [22,23]. Then, to avoid hyperglycemia and all its adverse effects on the mother and fetus, insulin therapy will become necessary [29]. The consequences of diabetic alterations may be significant for the development and function of the placenta [30]. Indeed, high maternal glucose provides microscopic changes in the placenta, such as chorangiosis, immature villi formation, syncytial knots, and zones of fibrosis [31]. The placental villi in pregnancies complicated by GDM are immature, this means that villous structures are less developed and mature in size [32]. This is associated with an increase in volume and a reduced count of terminal ramifications [33]. Another adaptation in GDM placentas is chorangiosis, which represents an excessive number of blood vessels in the villi [34]. The increased number of blood vessels of the chorangiosis should be considered a compensatory reaction to chronic hypoxia [34]. These attempts to deliver more oxygen to the fetus should result in an inefficient nutrient–gas exchange, which might be further deteriorated by these abnormal structures and functions of vessels. Syncytial knots have an increased frequency in GDM placentas [32,33]. These knots suggest that the placenta suffers from considerable physiological stress, probably due to the poor metabolic environment brought forth by hyperglycemia [32,33]. Moreover, these conditions may account for the significant increase in placental weight in GDM pregnancies [35]. Circulatory failure within the maternal–fetal system can be exacerbated by disorders related to metabolism and microcirculation, as well as by potential sclerosis and alteration of chorionic villi, uterine vessels, and the placenta in various forms of maternal diabetes [36]. Nevertheless, there are various degrees of severity associated with gestational diabetes [37]. The placenta in pregnant women with a controlled diet is not expected to differ much from normal morphology, but many studies revealed that even under careful diet control, several modifications could be observed [38]. Therefore, this systematic review aimed to establish the placenta’s microscopic and gross morphology difference in women with gestational diabetes who received only diet and exercise treatment.

## 2 Methods

### 2.1 Search strategy

Literature searches of Medline PubMed, EMBASE, the Cochrane Library, and Research Register (ClinicalTrial.gov) databases were conducted from the beginning to September 2024 with the key words “gestational diabetes,” “diet” or “exercise” and “placenta.” Two investigators (N.I. and R.M.) independently reviewed the titles, abstracts, and full-text articles. Additional articles were identified by searching the reference lists from the included studies. A third investigator (A.I.) checked search results and the articles included. Disagreements were resolved by consensus. The review methods were established at the beginning based on Preferred Reporting Items for Systematic Reviews and Meta-Analyses recommendations [39]. We registered the review to the PROSPERO site with protocol number CRD42024581621.

### 2.2 Eligibility criteria

The predefined inclusion criteria involved pregnant women with GDM controlled by diet and exercise (Class A1 following White classification of diabetes) [40]. Outcome measures were the gross or histopathologic placental anomalies examined in the included studies. Studies were not excluded on the basis of language or geographical location to ensure the widest set of findings relevant to the review topic. Studies were excluded if they were case reports, review articles, or metanalysis and if they examined animals’ placenta, if diabetes class was not specified.

### 2.3 Data extraction and analysis

Extracted data included characteristics of the population, diabetes class, and placental abnormalities. Two reviewers (N.I. and R.M.) performed the data extraction form, and a third reviewer inspected the data accuracy and completeness (A.I.). Given the different GDM diagnostic clinical practices adopted, data on the GDM criteria were also extracted. Given the substantive heterogeneity in the study methodology and the heterogeneity in data reported, abnormalities investigated, and population characteristics, it was not suitable to perform a quantitative meta-analysis. We assessed all included studies regarding potential conflicts of interest.

### 2.4 Outcome definitions

We categorized the published results of the gross and histological findings of the placenta results as presented by the authors. The studies included reported different gross and histological findings. Therefore, we extracted data concerning the placenta variables and reported findings of the variables mentioned in different columns in Tables 1 and 2.

**Table 1:** Macroscopic characteristic of placenta (mean ± SD or – CI 95%)

	Placenta weight (g)	Large diameter (cm)	Placenta thickness (cm)	Small diameter (cm)	Cord length (cm)
Arshad et al. [44]	590 ± 147.9	15.06 ± 2.41	2.84 ± 0.62	—	42.96 ± 7.4
Arshad et al. [45]	590 ± 147.9	15.06 ± 2.41	2.84 ± 0.62		42.96 ± 7.4
Arshad et al. [46]	579.10 ± 128	14.7 ± 2.34	2.65 ± 0.63		
Kapustin et al. [42]	619.7 (611.9–627.4)	20.7 (20.5–21)	3.52 (3.04–3.92)	22 (21.6–22.4)	—
Lao et al. [43]	634 ± 159	—	—	—	—
Liang et al. [48]	640 (590, 720)	—	2.99 ± 0.22	—	—
Nataly et al. [50]	514 ± 122	—	—	—	—
Kucuk and Doymaz [49]	694.8 ± 152.1	—	—	—	—

**Table 2:** Microscopic characteristic of placenta

	Chorangiosis	Infarction	Calcification	Villous fibrinoid necrosis	Syncytial knots	Fetal vascular malperfusion lesions
Arshad et al. [44]	—	—	—	2 (8%)	—	—
Arshad et al. [45]	13 (52%)	14 (56%)	10 (40%)	19 (76%)	14 (56%)	—
Arshad et al. [46]	14 (40%)	17 (48%)	13 (37%)	20 (57%)	17 (48%)	—
Kapustin et al. [42]	1260 (76%)	102 (6.2%)	582 (35%)	252 (15.2%)		—
Nataly et al. [50]	—	—	—	—	—	13 (17.8%)

### 2.5 Methodological quality assessment

We assessed the quality of the included studies using a modified version of the Newcastle–Ottawa scale [41], with five different domains reported in Table S1. Two authors (N.I. and R.M.) independently rated the study’s quality. Any disagreement was subsequently resolved by discussion or consultation with A.I.

## 3 Results

### 3.1 Study selection

**Figure 1:**
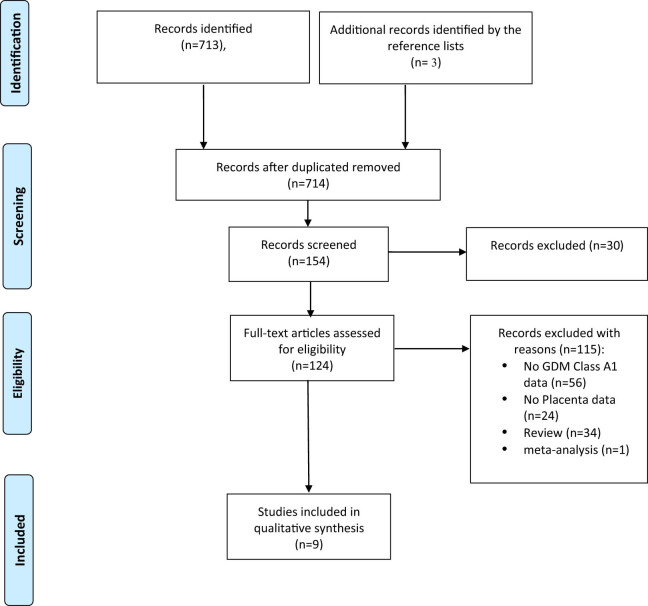
Characteristic of studies qualified for systematic review.

After removing records with no full text, duplicates, and wrong study designs (e.g., reviews), nine studies matched the inclusion criteria and were included in the systematic review [42–48]. Figure 1 shows the study selection.

### 3.2 Study characteristics

The countries where the studies were conducted, the publication year range, the studies’ design, the period of enrollment, and the total number of participants are summarized in Table 3. Overall, the publication years ranged from 1994 to 2020 [43,48]. The enrollment period ranged from 9 months to 36 months [44,49], two author did not specify the period [42,44] (Table 3). Two studies had a retrospective cohort study [42,47] and two a retrospective case–control study [43,48]; while three studies were prospective cohort studies (Table 3) [44–46,49,50]. Of these, three studies were conducted by Arshad et al. from Pakistan [44–46], two from China [43,48], one from Denmark [47], one from Russia [42], one from Israel [50], and one from Turkey [49] (Table 3). The total number of patients considered in the studies was 5,142. If we consider only the GDM in diet group, the number of patients included in this systematic review was 1,904 (Table 3).

**Table 3:** Characteristics of studies included

Study	Country	Study type	Period of enrollment	Total no. of patients	Patients included in the GDM-A1 group
Arshad et al. [44]	Pakistan	Prospective cohort study	9 months, unknown year	50 (initially, 69)	25 GDMA1 group (initially, 30)
Arshad et al. [45]	Pakistan	Prospective cohort study	From June 2010 to June 2011	87	30 GDMA1 group
Arshad et al. [46]	Pakistan	Prospective cohort study	From January 2018 to February 2019	77	35 GDMA1 group (initially 42; 7 lost during the follow-up)
Kapustin et al. [42]	Russia	Retrospective cohort study	Unknown	3,300	1,652 GDMA1 group
Lao 1996	China	Retrospective case–control study	1994	956	21 GDMA1 group
Thunbo et al. [47]	Denmark	Retrospective cohort study	From July 2015 to December 2016	52	8 GDMA1 group
Liang et al. [48]	China	Retrospective case–control study	From November 2018 to June 2020	60 (initially, 1,861)	30 GDMA1 group
Nataly et al. [50]	Israel	Prospective cohort study	From 2016 to 2019	256	73 GDMA1 group
Kucuk and Doymaz [49]	Turkey	Prospective cohort study	From May 2003 to June 2006	304	30 GDMA1 group

Abbreviations: GDMA1: gestational diabetes mellitus controlled with diet; OGTT: oral glucose tolerance test.

### 3.3 Risk of bias of included studies

Of these nine studies, six were considered to have a low risk of bias in at least three domains [42–46,50]. Three studies were considered to have high risk of bias [47–49]. Table S2 describes the detailed assessment of the risk of bias for each domain in each study.

### 3.4 Synthesis of the results

Considering the macroscopic characteristics of the placenta, eight studies evaluated the placental weight [42–46,48–50] and five placental thickness [42,44–46,48] (Table 1). Four studies included the large diameter of the placenta [42,44–46] (Table 1). One study did not report any macroscopic data [47]. Three studies evaluated the microscopic alterations of the placenta as chorangiosis, infarction, calcification, and villous fibrinoid necrosis [42,45,46]. Two studies analyzed the syncytial knots [45,46]. One reported the fetal vascular malperfusion lesions [50].

#### 3.4.1 Gross morphology of placenta

A total of 1,904 patients were included in this systematic review (Table 3). The main number of patients included arrived from the Kapustin et al.’s study (1,652 pregnant women) [42]. Seven studies described the gross morphological characteristics of the placentas from pregnancies complicated by GDM managed only by dietary control [42–46,48,50]. The mean placental weight of the diet control group ranged from 590 to 694 g. Arshad et al. compared the placenta of women with GDM managed by diet control with those requiring insulin therapy [44]. The placental weight was significantly higher in the insulin group versus the diet-controlled group (Table 1). Arshad et al. revealed that the placentae from GDM patients managed only with diet control were distinctly different as compared to normal pregnancy [45]. The study included a set of 30 cases of GDM patients whose blood sugar level was controlled with a restricted diet and regular exercise regimen [45]. The placental weight was increased in the placentae of the diet-controlled group compared with the control group but appeared to be less prominent when compared with GDM patients on medication such as metformin (Table 1) [45]. Arshad et al. compared the gross morphology of placentae from women with diet-controlled gestational diabetes with normal pregnancy [46]. They demonstrated an increase in placental weight in the GDMA1 group as compared to the control, *p* < 0.01 was recorded [46]. This suggested changes in the structure even under dietary control. However, placental weight and surface size did not show a statistical difference between the GDM group and the control group (Table 1) [46]. Kapustin et al., with a retrospective study, analyzed the placentae of women with GDM and managed by diet alone in the context of a larger cohort study [42]. The study involved a sample size of 3,300 placentas, thereby representing a comparison between different morphological characteristics related to different types of GDM, including GDM on diet. The placentae from women with diet-controlled GDM had an increased size compared to the control (mean placental weight, 619.7 g) [42]. However, these changes were less noticeable with respect to the insulin-treated groups. Lao et al. conducted a retrospective case–control study in which placentae from 478 pregnancies were analyzed, comparing the morphological placental characteristics in pregnant women with impaired glucose tolerance versus patients with diet-controlled GDM [43]. As this present study demonstrated, placentae from women with diet-controlled GDM were of increased size compared to the control group (mean placental weight was 634 ± 159 g) (Table 1) [43]. In a comparative study, Liang et al. explored the morphology of placentas in women with diet control GDM. The sample size consisted of 60 placentas: 30 from GDM patients and 30 from healthy controls [48]. It showed a significant difference in placental weight, volume, and thickness of the GDM group. In the case of mean placental weight, they recorded a value of 640 g for the GDM group, compared to only 410 g for the control group (Table 1) [48]. Thunbo et al. found a large placental weight, defined as greater than the 90th percentile in 37.5% of the Typ1 1 GDM cases and 55.6% of GDM insulin-treated versus 12.5% in the GDM in the diet control group [47]. Nataly et al. found higher rates of placentas above the 90th percentile in GDM treated with insulin compared to the GDMA1 group [50]. Kucuk observed a higher placental weight in the GDMA1 group compared to one abnormal value on 100 g oral glucose tolerance test.

#### 3.4.2 Microscopic alterations of placenta

Five studies quantify the microscopic alterations of the placenta in the GDM diet group. Arshad et al. evidenced only the 8% fibrinoid necrosis in placentas noted in patients with GDM with diet control [44]. Placentas from insulin-treated patients were seen with increased fibrinoid necrosis, which is interpreted as tissue damage or possibly impaired placental function. Other gross hemorrhages and gross lesions were found to occur in both groups, but frequently in the insulin-treated group [44]. In contrast, in 2016, Arshad compared the microscopic analysis of placentas from diet control GDM (Group B) versus a control group (Group A) and a metformin-treated group (Group C) [45]. The placentas from the diet-controlled GDM group (Group B) exhibited a higher incidence of villous immaturity (76%), chorangiosis (52%), infarction (56%), and syncytial knots (56%) compared to the control group [45]. In contrast, the placentas from the metformin-treated group (Group C) showed fewer of these abnormalities, with results being more similar to the normal control group [45]. In a recent study, Arshad et al. reported an increased villous immaturity (57%), chorangiosis (40%), fibrinoid necrosis (57%), and syncytial knots (48%) of the GDM diet group compared to the control group [46]. However, these findings had no statistical significance except for the syncytial knots prevalence, which was more frequent in the GDM group (*p* = 0.025) [46]. In the research of Kapustin et al., among the placentas of women with different forms of diabetes (type 1 DM, type 2 DM, and GDM), the increased deposition of fibrinoid (15%) in the sub-choral space dominated in GDM managed only by diet [42]. Calcification (35%) was more frequent in the placentas of women with T2DM and GDM. The degree of calcification varied but was significantly increased compared to controls [42]. Lao et al. did not provide any microscopic analysis [43]. Thunbo et al. examined the placentas from pregnancies complicated by GDM for microscopic pathology [47]. The results showed villous immaturity that was delayed in 25% of the cases of T1DM and 33.3% of GDM insulin-treated pregnancies but was absent in both GDM in diet control and normal pregnancies [47]. Liang et al. studied the microscopic analysis of placentas from women with GDM, and revealed an increased prevalence of immature villus, fibrinoid necrosis, calcification, and vascular thrombosis in the GDM group (*p* < 0.05) [48]. Nataly et al. evidenced a higher rate of placental fetal vascular malperfusion lesions in the GDMA1 groups with no placental differences in placental maternal vascular malperfusion lesions, acute inflammatory lesions, and chronic villitis [50].

## 4 Discussion

This systematic review focused exclusively on placental alterations in GDM in diet therapy and exercise changes. Although dietary management of GDM was expected to mitigate some adverse effects on placental morphology, our review found gross and microscopic morphology alterations [51,52]. An increased placental weight in the diet-controlled GDM group was demonstrated versus the control group (physiological pregnancies). These results suggest that some effects of mild hyperglycemia on placental growth may persist even with meticulous dietary control [53]. At the same time, insulin therapy results in a high placental weight compared to the GDMA1 group [54]. The increased placental weight in the diet-controlled GDM group might reflect placental inefficiencies that lead to anomalies, such as fetal overgrowth or other maternal adverse outcomes [55]. In addition, diet-controlled GDM placentas showed several pathological histological alterations, like villous immaturity, chorangiosis, and fibrinoid necrosis [56]. Different studies included in this review evidenced a higher incidence of villous immaturity, chorangiosis, infarction, and syncytial knots in the diet-controlled GDM population. Also, fibrinoid in the sub-choral space and calcification prevalence were higher in the GDM managed only by diet. These findings provide evidence against the previous opinion that diet and exercise can fully compensate for the effects of GDM on placental development [55]. Diet interventions are critical for maintaining blood glucose levels [57]. The placental morphology changes suggest that even mild hyperglycemia could impact the placenta. Moreover, our conclusions are not generalizable due to the several limitations. First, there is a great heterogeneity of the studies included. Considerable designs, population characteristics, and methodological differences are included in the studies. The retrospective nature of different included studies limits the long-term impacts of diet-controlled GDM on placental morphology evaluation. Inclusion of studies with high-risk bias reduces the generalizability of our findings. Fewer studies reported complete microscopic analyses. Variability in the diagnostic test for GDM and variability in diet performed further increase the population heterogeneity and complicate the interpretation of the results [58]. At least, pre-existing uterine pathologies, including uterine myoma or previous uterine surgeries, and their negative impact on placentation were not evaluated by the studies included [59–61]. These conditions could interfere with the placental maturation and increase the incidence of further abnormalities, such as villous immaturity and chorangiosis [62].

In contrast, some strengths are identified in our review: a systematic literature review is conducted with a strong protocol that adds credibility and completeness to the research taken into consideration we focus on GDM through diet control, a specific study area with a gap in the current literature. A total of 1,904 patients were included in our analysis. The participants, pregnant women, come from different countries, adding to the generalizability of our results. Finally, additional studies are needed to compare different diets and physical changes and their impact on the placental morphology and function. Our findings should alert obstetricians that even with diet-controlled GDM, persistent placental abnormalities may persist, and maternal–fetal surveillance should be performed. Specific gross morphological and microscopic evaluation of the placenta in GDM should be performed to understand the real diet implication on the fetoplacental unit. Chronic insufficiency due to chorioangiosis hypervascularization, pathological immaturity of villi, calcifications, and other disorders should be better investigated.

In conclusion, GDM patients who received diet and exercise treatment presented placenta’s gross and microscopic alterations. These alterations are less pronounced than in GDM patients receiving insulin therapy. Further research must confirm or rule out placental lesions and their association with maternal glycemia.

## Supplementary Material

Supplementary Table
