# Supplementary material for: Microscopic changes and gross morphology of placenta in women affected by gestational diabetes mellitus in dietary treatment: A systematic review
Source: Open Med (Wars). 2025 Feb 13;20(1):20251142. doi: 10.1515/med-2025-1142 (PMC11826244; doi:10.1515/med-2025-1142)
Supplement: Supplementary Table [file med-2025-1142-sm.pdf]

Supplementary material

Table S1: Modified Newcastle-Ottawa scoring items

(1) Study design and sample representativeness:
1 point: Study design involved a control group, GDMA1 sample size was greater than or equal to 100.
0 points: Uncontrolled study, sample size less than 100 participants.
(2) Sampling technique:
1 point: Patients recruited consecutively or randomly (randomization criteria clarified).
0 points: Potential convenience sampling or unspecified sampling technique.
(3) Description of the placental analysis technique, microscopic and macroscopic:
1 point: The authors provided a comprehensive description of the equipment, setting, and
adopted technique.
0 points: The study did not report adequate information on the placental evaluation technique.
(4) Quality of population description:
1 point: The study reported a clear description of the population with proper measures of dispersion (e.g., mean, standard deviation).
0 points: The study did not report a clear description of the population, incompletely reported
descriptive statistics or did not report measures of dispersion.
(5) Incomplete outcome data:
1 point: The study reported complete data about the placental histological abnormalities.
0 points: Selective data reporting cannot be excluded.

Abbreviations: GDMA1: gestational diabetes mellitus controlled with diet.
The individual components listed above are summed to generate a total modified Newcastle-Ottawa.
risk of bias score for each study. Total scores range from 0 to 5.
For the total score grouping, studies were judged to be of low risk of bias (≥3 points) or high risk of bias (<3 points).

Table S2: Risk of bias assessment of the 7 included studies

Author, Year	Study design and sample representativeness	Sampling technique	Description of the placental analysis technique	Quality of population description	Incomplete outcome data	Total score
R. V. Kapustin 2021 [1]	–	–	★	★	★	★★★
Arshad R. 2014 [2]	-	★	★	–	★	★★★
Arshad R. 2016 [3]	–	★	★	–	★★	★★★
Arshad R. 2023 [4]	–	★	★	–	★	★★★
Lao T. T. 1996	★	★	-	★	–	★★★
Thunbo M.Ø. 2018 [5]	–	★	–	★	–	★★
Xinyan L. 2023 [6]	–	★	–	–	–	★
Nataly F. 2022 [8]	–	★	★	★	★	★★★★
Kucuk M. 2009 [7]	–	★	–	★	–	★★

References

[1] Kapustin RV, Kopteyeva EV, Tral TG, Tolibova GK. Placental morphology in different types of diabetes mellitus. J Obstet Women's Dis. 2021;70(2):13–26.

[2] Arshad R, Karim N, Hasan JA. Effects of insulin on placental, fetal and maternal outcomes in gestational diabetes mellitus. Pak J Med Sci. 2014;30(2):240.

[3] Arshad R, Kanpurwala MA, Karim N, Hassan JA. Effects of diet and metformin on placental morphology in gestational diabetes mellitus. Pak J Med Sci. 2016;32(6):1522.

[4] Arshad R, Amir E, Pahore AK, Mustansar T. Histo-morphometric vicissitudes in diet-controlled diabetic placenta: A quantitative stereological model. Gynecology and Obstetrics. Clin Med. 2023;3(2):112–8.

[5] Thunbo MØ, Sinding M, Bogaard P, Korsager AS, Frøkjær JB, Østergaard LR, et al. Postpartum placental CT angiography in normal pregnancies and in those complicated by diabetes mellitus. Placenta. 2018;69:20–5.

[6] Liang X, Zhang J, Wang Y, Wu Y, Liu H, Feng W, et al. Comparative study of microvascular structural changes in the gestational diabetic placenta. Diabetes Vasc Dis Res. 2023;20(3):14791641231173627.

[7] Kucuk M, Doymaz F. Placental weight and placental weight-to-birth weight ratio are increased in diet-and exercise-treated gestational diabetes mellitus subjects but not in subjects with one abnormal value on 100-g oral glucose tolerance test. J Diabetes Complications. 2009;23(1):25–31.

[8] Nataly F, Hadas GH, Ohad G, Letizia S, Michal K. Is there a difference in placental pathology in pregnancies complicated with gestational diabetes A2 versus gestational diabetes A1, versus one abnormal value, on 100 gr glucose tolerance test? Placenta. 2022;120:60–4.
